# More age-care staff report helping care recipients following a brief depression awareness raising intervention

**DOI:** 10.1186/1472-6955-12-10

**Published:** 2013-04-05

**Authors:** Joanna Atkins, Sharon L Naismith, Georgina M Luscombe, Ian B Hickie

**Affiliations:** 1 Brain & Mind Research Institute, University of Sydney, 94 Mallett Street, Camperdown, NSW, 2050, Australia; 2 Clinical Research Unit, Brain & Mind Research Institute, University of Sydney, 94 Mallett Street, Camperdown, NSW, 2050, Australia; 3 School of Rural Health, and Adjunct Lecturer, Department of Obstetrics & Gynaecology, Sydney Medical School, University of Sydney, Sydney, Australia

**Keywords:** Depression, Older people, Intervention

## Abstract

**Background:**

Those working with elderly care recipients require a good working knowledge of depression and appropriate help giving responses. While it is important for age-care staff to recognize depression in care recipients it is also critical that they know the appropriate course of action to assist a care recipient who may be depressed. This study aims to determine the knowledge of age-care staff of appropriate help giving responses, their confidence in knowing what kind of assistance to provide and their actual likelihood of providing help to potentially depressed care recipients and to examine if these measures improve following an intervention training program.

**Methods:**

One hundred and two age-care staff were surveyed on their confidence in helping age-care recipients and on their knowledge of appropriate ways to provide assistance. Staff then participated in a two hour depression awareness raising intervention. The survey was repeated immediately following the training and again six months later.

**Results:**

Staff confidence in knowing how to provide assistance increased significantly subsequent to training and remained significantly improved at the six month follow up. In addition, a significantly higher proportion of staff reported helping care recipients at the six month follow up.

**Conclusions:**

This study highlights the potential of a brief staff training program to provide a cost effective means to improve staff self-confidence and increase the likelihood of staff providing assistance to depressed care recipients.

## Background

Those working closely with the elderly need to have a good level of awareness of depression because of its high incidence in elderly care recipients [1,2]. Despite these high rates, detection of depression by staff tends to be low, with different studies showing rates between 15 and 38% for nursing staff (depending on the assessment method used) and 62% for nursing home managers [3]. These low detection rates have been found in a variety of settings, including nursing homes [4] and home care settings [1].

Unlike general practitioners (GPs), age-care staff have regular ongoing contact with care recipients and are well placed to recognize symptoms that could be indicative of depression. Indeed, some data suggest that residents are more likely to disclose depressive symptoms to nursing home staff than to their GP [5]. Early intervention is important as it improves long term outcomes and reduces suffering [6], particularly since depression is a risk factor for dementia [7] and can lead to increased physical health problems and mortality [8,9]; high health service utilisation [10] and to decline in functional status [11]. Early intervention of depressive symptoms is also warranted since pharmacological and psychological treatments are effective [12].

Recognition rates of depression in older persons have typically been reported to be low [13] although more recent research indicates that the situation may be improving [14]. Recognition in this age group is particularly difficult because of the overlap of symptoms between depression and physical health problems [15,16] and between depression and dementia [17,18]).

While it is important for age-care staff to recognize depression in care recipients it is also critical that they know the appropriate course of action to assist a care recipient who may be depressed. Previous research has shown that age-care staff may not be aware of appropriate helping behaviours. For example, an Australian study [19] found that low care and community care staff were unsure of referral procedures, whether to notify a GP or mental health specialist and of what information they should provide. Another study [20] found that even if professional carers did make psychiatric referrals for depressed residents the reasons given usually did not relate to depression but to ‘behaviour problems’ or anxiety.

It is likely that a lack of training in depression awareness is responsible for low detection rates and lack of confidence in knowing what kind of assistance to provide. Studies have found rates of mental health training of between 7.8% and 40% [19,21,22]. The latter study also found that staff who had previous depression training had greater self-efficacy scores (that is ‘confidence in working with depressed older people,’ page 1291) but not greater knowledge scores and that personal carers had the least knowledge of late life depression compared to managers and nurses (including responding to depression). Other studies [21] have shown that nurses are no better at identifying depression than other staff members. In addition, length of service has also been shown to be unrelated to level of knowledge about depression [22,23].

Improving detection rates does not necessarily translate into improved management of depression in terms of help giving behaviours by staff. For example, a meta-analysis [24] found little or no impact of depression screening questionnaires on either the detection or management of depression by clinicians. A recent study [25] investigated staff knowledge and self-efficacy related to recognizing and responding to depression and whether training improved numbers of referrals for depression. While the authors did find significant improvements in knowledge and self-efficacy there were no significant increases in referrals for depression at follow up.

Investment in age-care staff training needs to be inexpensive and take account of the high staff turnover in age-care facilities. The present study looks at a brief staff training intervention which is easy for facilities to implement, inexpensive and consequently can be repeated on a regular basis to counter the effects of staff turnover.

While a number of studies have looked at staff training in depression recognition [26-28]; staff knowledge of depression [23,29] and confidence in managing depression [22,25]; to our knowledge, no other studies have explored unprompted knowledge of appropriate help giving behaviours or examined staff confidence in knowing how to respond to depression in high care environments. For this reason, the present study aims to extend previous research by focusing on staff self-efficacy and knowledge of appropriate help giving responses and their likelihood of providing assistance to potentially depressed care recipients. The study also examines those factors that influence confidence, knowledge and help giving behaviours including:

•Staff role (nursing staff versus personal carers, managers and general staff);

•Length of service;

•Previous training in the health field or mental health field;

•Education status.

The study included all levels of age-care, from institutional high care to at home community care (see below) and included all staff who were available to participate in the training program.

## Methods

### Sample

The sample consisted of 102 age-care staff, including managers (community care program co-ordinators and residential care managers), personal carers (including personal care assistants, direct carers and care services employees), nursing staff (registered nurses and assistants in nursing) and general staff (including office staff, recreational activities officers, and service staff) from five age-care settings that, according to the facility managers, did not have formalized procedures for the recognition and management of depression. The facilities included high care (one nursing home, n = 32 staff), low care (two hostels and a retirement village, n = 25 staff) and community care (home care services, n = 45 staff) in the Sydney Metropolitan area. In Australia, care recipients are assigned to high or low care status in accordance with the Aged Care Funding Instrument (ACFI) and assignment is based on the severity of scores on three domains: activities of daily living, behaviour, and complex health care (see http://www.health.gov.au/internet/main/publishing.nsf/Content/ageing-acfi-using-weightings.htm). Nursing home accommodation is provided for care recipients with high care needs and hostel accommodation is provided for care recipients with low care support needs who require some assistance with the demands of daily living, but who do not have complex ongoing care needs.

General staff and managers were included for both comparison purposes and because there may be occasions when they, particularly facility managers, are required to assist potentially depressed care recipients.

### Measures

Staff were surveyed on the following outcome measures:

1. Confidence in knowing how to assist depressed care recipients

Staff were asked: *How confident do you feel that you know how to assist one of your residents to get appropriate care if they are depressed?* They were asked to respond on a five point Likert scale, with ‘1’ being the least confident and ‘5’ the most confident.

2. Knowledge of appropriate helping responses

Staff were asked what they would do if a resident was showing symptoms of depression. Determination of the appropriateness of these unprompted helping responses was by consensus of three of the authors (one psychiatrist and two psychologists). Helping responses were coded according to whether they were considered appropriate, that is ‘evidence based’/ procedural (e.g. *counselling/therapy/CBT; discuss with GP/family/social worker*; *make appointment with health care professional*); or ‘non-specific’ (e.g. *be positive/cheerful*, *praise them*, *encourage pride in appearance*). Each *appropriate* response was given a score of ‘one’ and these scores were totaled to give an overall knowledge of appropriate help giving responses score.

3. Help giving behaviours

Staff were asked if they had helped care recipients in the last three months in any of six specific ways: *Suggest they try to get hold of self-help materials; Suggest they go to a GP/ doctor or other health professional; Assist them to make an appointment with their doctor; Suggest they go to a psychologist or other mental health professional; Go with them to see a doctor or health professional****;****Follow them up and make sure that they got professional help* (taken from a similar question in the *beyondblue* 2004 Community Survey [30]).

In addition, demographic information was collected including: gender, age, education level, length of service, professional training in the health field and professional training in the mental health field.

### Procedure

Staff responses were gathered at three time points: before training (BT), immediately after the training (AT) and six months after training (FU). BT data comprised information collected at enrollment (demographics and help giving behaviours) and immediately before commencement of the training (questions on confidence and knowledge related to help giving behaviours). The confidence and knowledge questions were repeated at the AT and FU time points and the help giving behaviour questions were repeated at FU (to provide staff a sufficient time period to demonstrate the effects of the training on actual practice).

### Ethics approval

Ethics approval to conduct the project was granted by the University of Sydney Human Research Ethics committee. Participants gave informed consent.

### Depression awareness training

The awareness training consisted of a two-hour presentation by the first author and covered the following areas: definition and types of depression, symptoms, prevalence, causes, risk and protective factors, susceptibility of the elderly, consequences of untreated depression, evidence-based treatments, appropriate helping behaviors, myths and facts, stigma, and where to get help. Staff also received a book on depression [31] and a booklet produced by the first author summarizing the information from the training session.

### Statistical analysis

Analyses were conducted using SPSS Version 19. Analyses examined differences in the outcome measures before and immediately after the intervention (BT versus AT) with the exception of data on the proportion of staff who provided help to depressed care recipients which were not collected at AT. To examine whether training effects were sustained, comparisons were also made between BT and FU.

Of the outcome measures, the variable measuring staff confidence was ordinal (Likert Scale 1–5), and the knowledge question was a continuous variable which was significantly skewed. Consequently the non-parametric Wilcoxon Signed Ranks test was used to assess differences between time points (for the sake of comparisons, mean data are presented in addition to medians and interquartile ranges). The proportions of staff at BT and FU who said they provided at least one of each of the six help giving behaviours were calculated and the proportions compared over time using the McNemar test for two-related samples.

The relationships between staff demographic variables (*role, length of service, education status, previous training in health/mental health*) and the two continuous outcome measures prior to training were explored using the Kruskal Wallis test followed by the Mann Whitney U test for pairwise comparisons. The relationship between staff demographic variables and the proportion of staff who provided assistance was measured using the chi square test.

Chi square analyses were used to compare demographic characteristics of staff members who were available at FU compared to those who were not (completers versus non-completers) with continuous variables (*age* and *length of service*) recoded into dichotomous categorical variables (age was recoded into *under 40 years/ 40 and above* and length of service was recoded into *up to three years/more than three years*). Chi square analyses were also used to assess differences at baseline between completers and non-completers on the categorical outcome variable *helped/didn’t help* for each of the six helping behaviours and the Mann Whitney U test was used to assess differences at baseline on the two continuous outcome variables (*Confidence in knowing how to assist depressed care recipients* and *Knowledge of appropriate helping responses*).

Where applicable, tests were two tailed and significance was set at a level of 0.05.

## Results

Surveys were returned by 102 staff (53.1% of the total staff employed) at BT and AT and by 51 staff at FU (50.0% of BT sample).

### Demographics

The majority of staff were female (89.8%), with a mean age of 47.0 years (SD 11.4), an education level of certificate or diploma and above (54.4%), with nursing staff most likely to have a higher level of education (75.0%), followed by general staff (50.0.%), carers (46.2%) and managers (25.0%). Staff had an average of 4.6 years (SD 5.4) service at the current facility. The majority of the staff had no professional training in the health field (60.6%) or mental health field (88.1%). Nurses were most likely to have had prior training in mental health (25.0%) and managers least likely (0%). Staff were employed as nurses (18.6%), personal carers (53.9%), general staff (20.56%), managers (4.9%) and two staff members did not identify their role (See Table 1).

**Table 1:** Staff demographics by role

**Characteristic**	**Nurse**	**Carer**	**Manager**	**General**
	**n = 19**	**n = 55**	**n = 5**	**n = 21**
**Female**% (n/N)	94.1 (16/17)	92.6 (50/54)	100.0 (5/5)	75.0 (15/20)
**Age (years)** Mean (SD)	47.3 (12.7)	46.7 (10.7)	51.2 (6.0)	46.5 (12.2)
**Education: certificate or diploma and above**% (n/N)	75.0 (12/16)	46.2 (12/26)	25.0 (1/4)	50.0 (10/20)
**Length of service (years)** Mean (SD)	3.6 (6.0)	4.3 (4.3)	5.6 (7.3)	5.8 (6.2)
**Professional training in the health field**% (n/N)	87.5 (14/16)	30.8 (8/26)	0/4	16.7 (3/18)
**Professional training in the mental health field**% (n/N)	25.0 (4/16)	11.5 (3/26)	0/4	5.3 (1/19)

N.B. not all participants answered all questions.

### Outcome measures

1. Confidence in knowing how to respond to a depressed care recipient

As shown in Table 2, there was a significant increase in the level of confidence in knowing how to respond immediately following the training (Z = −4.9, P < 0.001) and this increase remained significantly higher at FU (Z = −2.4, P = 0.016) indicating staff had maintained their improved level of confidence over this time period.

**Table 2:** Staff levels of confidence in knowing how to respond to a potentially depressed care recipient

	**N (BT)**	**BT**	**N (AT)**	**AT**	**N (FU)**	**FU**
		**Mean (SD)**		**Mean (SD)**		**Mean (SD)**
		**Median (Interquartile range)**		**Median (Interquartile range)**		**Median (Interquartile range)**
Confidence in knowing how to assist a depressed care recipient	101	3.5 (1.1)	98	4.1 (0.9)**	48	3.7 (0.9)*
		3 (1)		4 (1)		4 (1)
Number of appropriate responses identified	95	2.9 (1.7)	79	2.9 (1.8)	51	2.7 (1.9)
		3 (3)		2 (3)		2 (2)

Knowledge of appropriate responses before training (BT), after training (AT) and six month follow up (FU).

N.B. analyses compared BT vs AT and BT vs FU. Ns differ between variables due to missing data * P < 0.05**P < 0.001.

At BT nurses were significantly more confident than general staff (Z = −2.5, P = 0.016) but no more confident than personal carers or managers. Length of service was not related to confidence. There was a trend for those with a higher education status to have more confidence than those with a lower education status (with means of 3.6 and 3.1 respectively, P = 0.061). Those with training in the health field were significantly more confident than those without training, however there were no differences in confidence for those who had training in mental health compared to those who had not.

2. Knowledge of appropriate help giving responses

As shown in Table 2, knowledge of appropriate help giving responses did not increase significantly subsequent to training.

At BT, nurses did not name significantly more appropriate help giving responses than any of the other staff categories. There were no differences for education status, length of service or for training in the health field. Those with past training in the mental health field named significantly more correct responses than those without past training (a mean of 3.8 compared to a mean of 2.5, Z = −2.2, P = 0.032).

3. Help giving behaviors

As shown in Table 3, the percentage of staff who reported providing help to depressed care recipients increased significantly for four of the six help giving behaviors between BT and FU.

**Table 3:** Proportion of staff who reported providing assistance before training (BT) and at six month follow up (FU)

	**% of staff who helped**		
	**BT (n = 102)**	**FU (n = 48)**	**BT vs FU**
	**% (n)**	**% (n)**	**P**
Suggest they try to get hold of self-help materials	4.9 (5)	14.6 (7)	0.289
Suggest they go to a GP/ doctor or other health professional	2.9 (3)	37.5 (18)	<0.001
Assist them to make an appointment with their doctor	2.0 (2)	33.3 (16)	<0.001
Suggest they go to a psychologist or other mental health professional	2.9 (3)	12.5 (6)	0.289
Go with them to see a doctor or health professional	1.0 (1)	12.5 (6)	0.031
Follow them up and make sure that they got professional help	2.0 (2)	31.3 (15)	0.001

BT, before training; FU, follow up.

At BT there were no differences in likelihood of helping in terms of role, length of service, training in the health field or education status but those with prior training in mental health were significantly more likely to provide help for five of the six helping behaviours (all but: *follow them up and make sure they got help*; P = 0.016, 0.003, <0.001, 0.003, 0.007, 0.094 respectively).

### Staff completers versus non-completers

Staff with higher levels of education were significantly less likely to have FU data than those with lower levels of education (χ^2^ = 4.8, df = 1, P = 0.028). There were no other demographic differences or differences at baseline on the three outcome variables between completers and non-completers.

## Discussion

The results of this study suggest that a brief depression awareness training program for age-care facility staff is associated with increased confidence in knowing how to help age-care recipients who may be depressed and this change was sustained at the six month follow up. There was also a significant increase at follow up in the proportion of staff who said they provided help to care recipients for four of the six helping behaviours that were examined. Previous research [22] has highlighted that a lack of self-efficacy is a major barrier to age-care staff in recognizing and responding to depression in care recipients. Self-efficacy has been identified as an important factor in the likelihood of staff providing assistance for potentially depressed care recipients. For example, a recent study [32] found that self-efficacy was linked to improved knowledge about depression, care staff taking time to listen to and discuss depression with age-care recipients, and to a greater likelihood of communicating with other staff about depression in age-care recipients. It appears that increasing confidence levels leads to an increase in helping behaviours possibly by making it more likely that staff will act on the knowledge that they have when they were not confident to do so beforehand. It is therefore important that age-care facilities explore ways to increase staff confidence such as by using the training program outlined in this study.

In the present study, prior to training, the nursing staff reported that they were not significantly better at identifying appropriate responses to care recipients with depression than other staff and neither were they more confident in knowing how to respond. This is similar to Bagley et al., 2000 (op cit). In addition, nurses were no more likely than other staff members to help care recipients who were potentially depressed. If this is an accurate indication of their actual clinical responses this would be of concern as it is likely to be the nurses’ role to liaise with GPs and ensure that care recipients receive help. In the current study all of the nurses surveyed worked in high care where they would have a high level of exposure to care recipients with depression so it is of considerable concern that they did not have either greater knowledge of appropriate responses to depression or were no more likely to help than other groups of staff.

Prior to the training no relationship was found between length of service and any of the three outcome measures, which suggests that experience alone is not sufficient for age-care staff to develop a good working knowledge regarding assisting care recipients with possible depression. This finding is consistent with other studies that have shown no relationship between length of service and knowledge related to depression [22,23]. However it should be noted that the present study only examined length of service in the current position and not overall experience in age-care settings.

Those with previous training in the health field showed significantly higher confidence in knowing how to respond to depression than those without such training but they did not have greater levels of knowledge of appropriate ways of assisting care recipients with depressive symptoms and were no more likely to provide assistance. In contrast, while those with previous training in the mental health field could name significantly more appropriate ways of providing assistance to depressed care recipients and were also significantly more likely to provide help, they were no more confident than those without. This may be a reflection of the type of training they had received, which somehow failed to improve confidence. In contrast, the Davison et al. study (op cit) found that those with prior training in mental health had greater confidence but not greater knowledge to those without prior training. More research into the relationship between confidence and knowledge is warranted and into how each affect the other.

Prior to training, there was a trend (P = 0.061) for those with an education status of certificate/diploma and above to have greater confidence in knowing how to respond to depressed care recipients but they did not have greater knowledge of appropriate ways to respond and were no more likely to provide assistance than those with a lower education status. It is of concern that in the current study, managers were least likely to have an education status of certificate/diploma level and above and none of them reported having had previous training in either the health or mental health fields. Managers need to have good awareness of mental health in the elderly so they can direct staff in appropriate ways of responding. However it should be noted that the size of this group was extremely small.

Age-care staff work in a busy and challenging environment and care solutions need to be practical and not add to already heavy workloads. A study of hospital nurses in Hong Kong [33] found the second most common reason stated for not assessing patients for depression was lack of time (after lack of knowledge or skills). It is also important that depression be treated in the wider context of other chronic health conditions which are typical of elderly health care recipients [34,35].

There are a number of limitations in the present study. For example, this was not a randomized controlled trial but a single group pre-test post-test design and therefore because there was no comparison group any changes identified in outcome measures may be due to historical, maturation and testing effects. The study needs to be repeated with a control group for an accurate assessment of the benefits of the intervention. The measures used for help giving behaviors were based on staff self-report and recall rather than on objective, prospectively collected measures. It is possible that staff may have exaggerated their helping behaviours to appear depression-aware. However, the proportion of staff who said they provided assistance for each of the helping behaviours was extremely low, so it seems unlikely that numbers were inflated. Although staff in the current study may not have endorsed the six help giving behaviours included in the survey that does not mean that they did not provide some other kind of help for care recipients such as listening to them or recording their concerns in the care recipients’ notes. However, as the facilities used in the current study did not have formalized procedures for responding to depression some measures were needed for assessing helping behaviours. It is also not necessarily the case that the helping behaviour measures used in the current research would be available options for all the staff in all of the age-care facilities investigated which further highlights the need for formalized procedures to assist care recipients who may be depressed and for staff to be aware of and act upon these procedures. More appropriate measures may be determined in future research. In addition, only slightly more than half of staff returned surveys at baseline; a high percentage of staff were lost from the sample at FU, mainly due to high turnover of staff at the participating facilities; and missing demographic data (particularly for carers) at baseline means that results must be interpreted with caution. Those with a higher education status were less likely to have follow up data. These factors and the sampling of staff from only one geographic location limit the external validity of the results.

More research is needed to identify barriers to providing care to age-care recipients with depression, to find the best methods for providing training for staff to assist information retention; to understand the relationship between confidence and knowledge; to increase staff confidence; and to facilitate moving from a theoretical understanding to a practical application of knowledge by staff in their day to day work.

## Conclusion

Clearly there is a need for high quality training in depression recognition and management in age-care settings. The present study highlights the potential of a brief training program to increase staff self-efficacy and the number of staff who provide assistance to potentially depressed age-care recipients. The intervention is inexpensive and can be implemented on a regular basis to counter the effects of high staff turnover in age-care facilities.

## Competing interests

The authors declare that they have no competing interests.

## Authors’ contributions

JA helped with the design of the survey, conducted the data collection and staff training, performed the data analyses and wrote the paper. GML assisted with design of the survey, advised on statistical analysis of the data and assisted with revision of the paper. IBH and SLN supervised the research and assisted with revision of the paper. All authors read and approved the final manuscript.

## Pre-publication history

The pre-publication history for this paper can be accessed here:

http://www.biomedcentral.com/1472-6955/12/10/prepub
